# Comment on “Access to Abortion After Dobbs v. Jackson Women’s Health Organization: Advocacy and a Call to Action for the Profession of Psychiatry”

**DOI:** 10.1007/s40596-023-01901-7

**Published:** 2023-11-30

**Authors:** Mary E. Woesner, Scott M. Wilson

**Affiliations:** Albert Einstein College of Medicine, Bronx, NY USA

To the Editor:

We read with great interest the editorial by John Coverdale et al. on defining a role for academic psychiatry following the Dobbs decision [1]. In their editorial, the authors request that the field increase its commitment to those with mental illness and other vulnerable groups living in states with abortion bans, in part, by identifying those at risk of unwanted pregnancies.

One of us is a psychiatrist with a long history of working with patients with serious mental illness (SMI) and teaching this topic at the Albert Einstein College of Medicine; the other author is a medical student whose clerkship was in the setting of long-term, inpatient management of SMI. We are dedicated to improving the welfare of this population and feel it is important to respond to this call for action.

As of this writing, numerous states have imposed restrictions on abortion, including several that have implemented near outright bans. These limitations vary by state. In a few areas, abortions are prohibited after 6 weeks, a time when many women are unaware of their pregnancy. These stringent regulations can make it challenging to identify pregnancies in women with SMI early enough to provide timely and appropriate medical care, including medical and surgical abortion services for unwanted or high-risk pregnancy. Hence, there is an urgent need to identify factors that make individuals susceptible to unplanned pregnancy. There is considerable intersectionality between women with SMI and the vulnerable populations discussed by Coverdale et al., since this spectrum of conditions is more prevalent in people exposed to severe and traumatic life events [2]. These women include victims of all forms of sexual violence, those from lower socio-economic groups, and those with cognitive and neuropsychological deficits. These factors, along with conditions that impede access to medical care, can be identified and flagged.

The symptoms of mental illness also increase the risk of unwanted pregnancies among patients with SMI. A common belief about those with SMI is that they have diminished sexual desire and activity. However, cross-sectional studies have found a significantly higher prevalence of unwanted pregnancies and elective abortions among patients with major mental illness, including major depressive disorder, schizophrenia, and bipolar disorders I and II, relative to control populations [3]. During the HIV pandemic, there were numerous investigations of the relationship between psychiatric symptoms and high-risk sexual behavior. One cross-sectional birth cohort study found that, “young adults diagnosed with substance dependence, schizophrenia spectrum, depressive, manic and antisocial disorders were more likely to engage in risky sexual intercourse, contract sexually transmitted diseases, and have sexual intercourse at an early age” [4].

We agree with Coverdale et al. [1] that a “key first step” to prevent unwanted pregnancy is to conduct a thorough reproductive and sexual history in preparation for discussion of contraception. However, a typical reproductive and sexual history may not elicit risk factors for unwanted pregnancy specific to women with SMI. It may not include questions regarding symptoms and illness-related issues that increase the risk of sexual activity or unprotected sex. Episodes of mood elevation with concomitant grandiosity, hypersexuality, and impaired judgment in hypomanic and manic patients, as well as acute psychotic episodes and negative symptoms in patients with schizophrenia, may increase their vulnerability to sexual exploitation.

Follow-up can prove difficult for women with SMI, after discharge into the outpatient setting. A potential solution is to refer women of childbearing age with SMI—who have been identified as high-risk for unprotected sex and unplanned pregnancy—to an Assertive Community Treatment (ACT) team. This is a multidisciplinary team for psychiatric patients with high physical and/or behavioral health risk who do not attend outpatient visits. It provides individualized services by going into the community or the client’s home and can integrate medical services with psychiatric care. The team could identify and treat high-risk symptoms and educate patients and families on contraception. One of us has written about an ACT team that facilitated prenatal and obstetric care for a woman with manic symptoms due to Graves’ disease [5].

Sexual history-taking remains an undertaught subject across specialties and there is a lack of educational integration between psychiatric and obstetrics and gynecology (OB/GYN) programs.

To address concerns specific to patients with SMI, we advocate for an expanded curriculum for OB/GYN and psychiatry residents. Such training would also apply to medical students and mental health professionals working at psychiatric facilities. The curriculum would take a comprehensive approach, teaching participants how to elicit patient histories emphasizing social circumstances, comorbid diagnoses, and psychiatric symptoms that increase the risk for unprotected sex and unwanted pregnancy. High-risk patients would need to be identified, treated, and provided counseling about specific symptoms that increase their risk. The curriculum would further educate learners on running psychoeducational groups, used to teach families and caregivers to identify and report any increase in high-risk symptoms in the outpatient setting.

In cases where patients are noncompliant with clinic visits, the program would explore the utilization of an ACT team. This team would assess the feasibility of employing telemedicine and home visits to ensure high-risk patients receive the care they require.

Finally, in conjunction with the above, the program would incorporate contraceptive education for patients and their families. This would include emergency contraception, such as levonorgestrel and intrauterine devices, and medical abortifacients like mifepristone and/or misoprostol, if available in the state.

Psychiatrists have a long history of supporting the underprivileged and the stigmatized. Regrettably, the Dobbs decision diminishes the status and autonomy of women and has major implications for those living with SMI. Coverdale and colleagues impel us to counter the limitations placed on abortion access by the Dobbs decision. Going forward, we will need to protect the interests of patients with SMI who are most vulnerable and teach others to do so.
